# Understanding what drives schools to adopt effective school-based nutrition programs: a cross-sectional study of barriers and facilitators

**DOI:** 10.1093/tbm/ibaf079

**Published:** 2025-11-25

**Authors:** Demi Herdegen, Jannah Jones, Courtney Barnes, Nicole Nathan, Katie Robertson, Anna Rayward, Alison Brown, Molly Parkinson, Lisa Janssen, Stephanie Mantach, Elise Porter, Jessica Zorba, Julie Hunter, Aimee Mitchell, Christophe Lecathelinais, Luke Wolfenden, Rachel Sutherland

**Affiliations:** Hunter New England Population Health, Hunter New England Local Health District, Wallsend, NSW, Australia; School of Medicine and Public Health, University of Newcastle, Callaghan, NSW, Australia; Population Health Research Program, Hunter Medical Research Institute, New Lambton Heights, NSW, Australia; National Centre of Implementation Science, University of Newcastle, Callaghan, NSW, Australia; Hunter New England Population Health, Hunter New England Local Health District, Wallsend, NSW, Australia; School of Medicine and Public Health, University of Newcastle, Callaghan, NSW, Australia; Population Health Research Program, Hunter Medical Research Institute, New Lambton Heights, NSW, Australia; National Centre of Implementation Science, University of Newcastle, Callaghan, NSW, Australia; Hunter New England Population Health, Hunter New England Local Health District, Wallsend, NSW, Australia; School of Medicine and Public Health, University of Newcastle, Callaghan, NSW, Australia; Population Health Research Program, Hunter Medical Research Institute, New Lambton Heights, NSW, Australia; National Centre of Implementation Science, University of Newcastle, Callaghan, NSW, Australia; Hunter New England Population Health, Hunter New England Local Health District, Wallsend, NSW, Australia; School of Medicine and Public Health, University of Newcastle, Callaghan, NSW, Australia; Population Health Research Program, Hunter Medical Research Institute, New Lambton Heights, NSW, Australia; National Centre of Implementation Science, University of Newcastle, Callaghan, NSW, Australia; Hunter New England Population Health, Hunter New England Local Health District, Wallsend, NSW, Australia; Population Health Research Program, Hunter Medical Research Institute, New Lambton Heights, NSW, Australia; National Centre of Implementation Science, University of Newcastle, Callaghan, NSW, Australia; Hunter New England Population Health, Hunter New England Local Health District, Wallsend, NSW, Australia; School of Medicine and Public Health, University of Newcastle, Callaghan, NSW, Australia; Population Health Research Program, Hunter Medical Research Institute, New Lambton Heights, NSW, Australia; National Centre of Implementation Science, University of Newcastle, Callaghan, NSW, Australia; Hunter New England Population Health, Hunter New England Local Health District, Wallsend, NSW, Australia; School of Medicine and Public Health, University of Newcastle, Callaghan, NSW, Australia; Population Health Research Program, Hunter Medical Research Institute, New Lambton Heights, NSW, Australia; National Centre of Implementation Science, University of Newcastle, Callaghan, NSW, Australia; School of Medicine and Public Health, University of Newcastle, Callaghan, NSW, Australia; Population Health Research Program, Hunter Medical Research Institute, New Lambton Heights, NSW, Australia; National Centre of Implementation Science, University of Newcastle, Callaghan, NSW, Australia; Hunter New England Population Health, Hunter New England Local Health District, Wallsend, NSW, Australia; School of Medicine and Public Health, University of Newcastle, Callaghan, NSW, Australia; Population Health Research Program, Hunter Medical Research Institute, New Lambton Heights, NSW, Australia; National Centre of Implementation Science, University of Newcastle, Callaghan, NSW, Australia; Hunter New England Population Health, Hunter New England Local Health District, Wallsend, NSW, Australia; School of Medicine and Public Health, University of Newcastle, Callaghan, NSW, Australia; Population Health Research Program, Hunter Medical Research Institute, New Lambton Heights, NSW, Australia; National Centre of Implementation Science, University of Newcastle, Callaghan, NSW, Australia; Hunter New England Population Health, Hunter New England Local Health District, Wallsend, NSW, Australia; Population Health Research Program, Hunter Medical Research Institute, New Lambton Heights, NSW, Australia; Hunter New England Population Health, Hunter New England Local Health District, Wallsend, NSW, Australia; Population Health Research Program, Hunter Medical Research Institute, New Lambton Heights, NSW, Australia; Hunter New England Population Health, Hunter New England Local Health District, Wallsend, NSW, Australia; Population Health Research Program, Hunter Medical Research Institute, New Lambton Heights, NSW, Australia; School of Medicine and Public Health, University of Newcastle, Callaghan, NSW, Australia; Population Health Research Program, Hunter Medical Research Institute, New Lambton Heights, NSW, Australia; National Centre of Implementation Science, University of Newcastle, Callaghan, NSW, Australia; Hunter New England Population Health, Hunter New England Local Health District, Wallsend, NSW, Australia; National Centre of Implementation Science, University of Newcastle, Callaghan, NSW, Australia; Hunter New England Population Health, Hunter New England Local Health District, Wallsend, NSW, Australia; School of Medicine and Public Health, University of Newcastle, Callaghan, NSW, Australia; Population Health Research Program, Hunter Medical Research Institute, New Lambton Heights, NSW, Australia; National Centre of Implementation Science, University of Newcastle, Callaghan, NSW, Australia; Hunter New England Population Health, Hunter New England Local Health District, Wallsend, NSW, Australia; School of Medicine and Public Health, University of Newcastle, Callaghan, NSW, Australia; Population Health Research Program, Hunter Medical Research Institute, New Lambton Heights, NSW, Australia; National Centre of Implementation Science, University of Newcastle, Callaghan, NSW, Australia

**Keywords:** implementation science, child nutrition sciences, public health, diet

## Abstract

**Background:**

Effective school-based nutrition programs can improve children’s diet and achieve population-level health outcomes if implemented at scale.

**Purpose:**

This study aimed to (i) examine the determinants (i.e. the barriers and facilitators) to the adoption of *SWAP IT*, an effective school-based nutrition program, and (ii) examine associations between these determinants and school characteristics, including school size, socioeconomic status (SES), and geolocation.

**Methods:**

A cross-sectional study was conducted with one key decision maker (e.g. principals, assistant principals, and health and wellbeing officers) from primary schools across New South Wales (NSW), Australia. Descriptive statistics and logistic regression were used to identify determinants of *SWAP IT* adoption and examine associations with school characteristics.

**Results:**

Key decision makers who responded (*n* = 160; 47% participation rate) most frequently reported barriers included “expected workload for staff” (*n* = 52, 33%), and the “perception that parents and carers do not think it is the school’s place to provide this information” (*n* = 31, 19%). The most frequently identified facilitators were to “keep the program free” (*n* = 60, 38%), and “show evidence that SWAP IT supports the development of healthy habits in children” (*n* = 18, 11%). Significant associations were found between barriers and the following school characteristics: schools in higher SES areas were more likely to report the “expected workload for staff” (OR: 2.6; 95% CI: 1.3–5.2; *P *= .006), while schools in lower SES areas more often reported the “perception that parents and carers do not think it is the school’s place to provide this information” (OR 2.4; 95% CI: 1.0–5.6; *P = *.04). No significant associations were found between school characteristics and reported facilitators.

**Conclusion:**

Barriers to the adoption of school-based nutrition programs can differ by school characteristics, highlighting the importance of considering these factors when scaling up such programs to ensure equitable adoption.

**Clinical Trial information:**

The Clinical Trials Registration #ACTRN12623000145606

## Teaser text

Understanding what drives key decision makers from schools to adopt effective school-based nutrition programs can inform scale-up approaches and ensure equitable population-level health impacts.

Implications **Practice:** Identifying adoption determinants can facilitate a successful and equitable scale-up, leading to greater uptake of school-based nutrition programs and improved population-level health outcomes.

**Policy:** Identifying the barriers and facilitators to the adoption of effective school-based nutrition programs can provide insights to policymakers and can be used to enhance the uptake of similar health promotion programs.

**Research:** These findings offer valuable insights into the contextual factors influencing program adoption, and future research should continue to assess these determinants during scale-up planning to enhance program success.

## Background

Chronic diseases represent a major health burden globally, accounting for 75% of annual mortality [1]. An unhealthy diet, including an excess consumption of foods high in saturated fat and sodium (known as discretionary foods) and low in fruits and vegetables, is one of the top four leading risk factors contributing to the development of chronic diseases such as type 2 diabetes, cardiovascular disease, stroke, and some cancers [1]. Ensuring that healthy dietary behaviors develop during childhood is critical, as these behaviors are likely to track into adulthood [2] and, therefore, reduce the risk of future chronic disease [3]. However, the current dietary intake of children is not consistent with global and national dietary guidelines [4]. For example, only 4.3% of Australian children aged 2–17 years meet the national recommended vegetable intake per day and consume an average of 5.1 servings of discretionary foods (e.g. foods high in saturated fat, sodium, and added sugar) each day [5, 6]. This is also reflected in the food consumed by primary school-aged students at school, with almost 40% of the total energy in lunchboxes coming from discretionary foods [7]. As such, schools are an ideal setting for implementing programs that promote healthy eating among children [8]. They provide access to a large, diverse group of children from various demographic to socioeconomic backgrounds for extended periods each day [8–10]. Additionally, children consume more than one-third of their daily dietary intake while at school [11].

The World Health Organization Health Promoting Schools (HPS) framework recommends a whole school approach to school-based nutrition programs by targeting the school curriculum (i.e. teaching), environment (i.e. policies and procedures), and partnerships (i.e. families and community) [12]. Systematic reviews have shown that school-based programs aimed at improving children’s nutritional intake are effective when implemented on a small scale, but few have been tested or demonstrated success on a larger scale [13, 14]. Previously identified school-based nutrition programs have included interventions targeting food provision through school canteens, as well as programs focused on teaching education [15–17]. Evidence-based school nutrition programs have the potential to achieve a population-level impact and reduce the burden of chronic disease. However, to achieve such an impact, they must be implemented at scale [18]. The World Health Organization (WHO) defines scale-up as “the deliberate effort to increase the impact of successfully tested health interventions to benefit more people and foster policy and program development on a lasting basis” [19].

Identifying the determinants of widespread adoption, including barriers and facilitators, is a critical step to ensure successful scale-up of health programs [20–22]. These determinants may include factors such as capacity, cost and resources required to implement, and are used to inform the selection of evidence-based scale-up strategies in order to maximize the adoption of effective health programs [23]. Despite this, scale-up determinants are rarely assessed during the planning phase (prior to scaling up), particularly regarding school-based nutrition programs [24–26]. Identifying the determinants influencing the adoption of school-based nutrition programs during the scale-up planning phase allows for the selection of theoretically based scale-up strategies to increase the likelihood of adoption at scale [20].

Understanding whether these determinants vary based on school characteristics can help determine if scale-up strategies require tailoring or if a universal approach is appropriate. While previous studies offer insights into the determinants of school-based nutrition programs focused on school nutrition policies and guidelines [27, 28], few provide insights into the barriers and facilitators to schools’ adoption of nutrition education programs delivered directly to parents, such as SWAP IT.

Inequalities have been shown to exist in the prevalence of healthy dietary intake, particularly in areas of lower socioeconomic status (SES) [29]. Families in rural and remote locations generally have lower incomes and face a higher risk of developing chronic diseases [30]. This is also reflected in school populations, with schools located in lower socioeconomic and regional or remote areas reporting higher prevalence of poor dietary intake [30, 31]. School characteristics, including socio-economic status, geolocation, and school size, have been identified as key factors influencing the adherence and adoption of nutrition-based health promotion programs [32]. Ensuring health equity is a key component of the World Health Organization HPS framework [9]. However, currently, few studies regarding school-based nutrition programs provide insight into potential associations between determinants of adoption and school characteristics such as SES, geolocation, and school size [32]. Identifying whether determinants differ by these characteristics can support the design of strategies and help mitigate the risk that efforts to scale up school-based programs exacerbate existing inequities.

SWAP IT is an effective school-based nutrition program aimed at improving primary school-aged students’ dietary intake by reducing discretionary foods packed in lunchboxes and aligning lunchbox contents with Australian national dietary guidelines for children aged 5–12 years [33, 34]. SWAP IT differs from other school-based nutrition programs as it encompasses all three components of the World Health Organization Health Promoting Schools framework, with the primary component delivered directly to parents and caregivers. The program has been rigorously evaluated through multiple randomized controlled trials (RCTs) [33, 34], cost-effectiveness studies [35], and scalability assessments, which have found the program to be effective, scalable, acceptable to parents, carers and schools, and strongly aligned with school contextual factors (e.g. staffing capacity and school priorities) [34]. Scalability assessments help determine whether a program is suitable for scale-up by providing a structure for working through key considerations [36]. Once the scalability of a program is established, the next recommended step is to identify key barriers and facilitators to adoption [20]. This information is critical to guide the development and potential tailoring of strategies to facilitate broader dissemination.

Therefore, this study aimed to: (i) examine the determinants (i.e. the barriers and facilitators) to the adoption of an effective, school-based nutrition program (“SWAP IT”) and (ii) examine the potential associations between the identified barriers and facilitators, and school characteristics (including school size, SES, and geolocation).

## Methods

### Study design and ethics approval

A cross-sectional design was employed for the conduct of this study. This study has been reported in accordance with the strengthening the reporting of observational studies in epidemiology (STROBE) checklist [37].

### Sample

The sampling frame included schools that catered for children aged 5–12 years, specifically primary schools (which includes children aged 5–12 years) and combined schools (which include both children aged 5–12 years and those aged 12–18 years), located across 11 NSW Local Health Districts (LHDs) that had partnered with the research team to conduct a broader RCT to scale-up SWAP IT (ACTRN12623000145606). The list of potentially eligible schools was sourced from the publicly accessible Australian Curriculum, Assessment and Reporting Authority (ACARA) database in February 2023. The same eligibility criteria as for the aforementioned RCT, aimed at increasing adoption of SWAP IT, were applied to this cross-sectional study. To be eligible, schools were required to be from either the NSW Department of Education (Government) or the Independent sectors, be current users of a school-parent communication platform, i.e. used to automate the delivery of SWAP IT (Audiri) and not have previously implemented SWAP IT in the past 12 months. Schools that catered exclusively for children requiring specialist care, such as those providing disability support and feeding assistance, were excluded due to their distinct student needs. Secondary schools were also excluded, as they do not enroll children aged 5–12 years, which is the target age group for the SWAP IT program.

### Recruitment procedures

Principals of all eligible schools were invited to participate in the study via email, which contained a study information statement and a link to an online survey. Principals were instructed to provide a response from one key decision maker at the school—either themselves or a delegate (e.g. assistant principal or health and wellbeing officer)—who oversaw the implementation of programs such as SWAP IT. Those who did not complete the survey within one week of the initial email invitation received up to three reminders via telephone or email, inviting completion of the survey either online or via telephone with a trained research assistant.

### Data collection and measures

#### School characteristics

School characteristics, including school sector (Independent, Government), postcode, geolocation, and number of student enrollments, were obtained from the ACARA database. Schools were categorized as “small” (<264 student enrolments) or “large” (≥264 student enrollments) based on the median number of student enrollments in the broader RCT trial sample. School postcodes were used to classify schools as being located in higher SES or lower SES areas as per the 2016 Socioeconomic Index for Australia (SEIFA) [38]. School postcodes ranked in the top 50% of NSW were classified as higher SES, and the bottom 50% of postcodes as lower SES. School postcode was also used to classify school geolocation (i.e. geographic location) as “rural/regional” (very remote, remote, outer regional, inner regional) or “major cities” based on the Australian Statistical Geography Standard [39].

#### Barriers and facilitators of the adoption of SWAP IT

After being provided with a brief description of SWAP IT, participants were asked to select their primary barrier and facilitator to the adoption of SWAP IT from a predetermined list. The list contained 13 potential barriers and 14 potential facilitators. The survey also included an open-text response option labelled “other” for both barriers and facilitators, allowing respondents to identify additional determinants not already listed. The list was developed based on findings from previous qualitative and quantitative studies conducted by the research team [34], as well as insights from published literature and exploration of scale-up theoretical frameworks [40]. The list was reviewed by a multidisciplinary advisory group comprised of experts in school-based health promotion programs, public health nutrition, the education sector, implementation science, and parents prior to finalization. Anecdotal data collected from advisory group members, whose role is to support schools in implementing health promotion programs, was also used to refine the list further.

### Analysis

Analyses were conducted using SAS, version 9.4 (SAS Institute) [41]. Descriptive statistics, including means, frequencies, and proportions, were used to summarize school characteristics, as well as barriers and facilitators to school adoption of SWAP IT. Simple logistic regression models were used to examine associations between each of the three most frequently reported barriers and facilitators, and each school characteristic (school size, SES, and geolocation), resulting in a total of 18 models. Assumptions relevant to logistic regression were considered and met, including appropriate binary outcomes, independence of observations, and adequate events per variable (10 or more). Each model contained a single predictor, removing multicollinearity concerns. Statistical significance was set at *P* *<* .05 for all tests. The responses categorized as “other” were analyzed and either classified into additional barrier and facilitator categories or incorporated within existing response options. School SES, size, and geolocation were selected for analysis because of their associations with the prevalence of childhood overweight and obesity [42].

## Results

### Characteristics of participants

Among the 342 eligible schools, 160 (47%) participated in the survey. Survey responses were collected between February 2023 and April 2023. The characteristics of the participating schools were representative of the broader RCT sample. The key decision maker at the school, as identified by principals, was primarily the principal (*n* = 88, 55%), followed by assistant or deputy principals (*n* = 48, 30%) and health and wellbeing coordinators (*n* = 12, 7.5%). The participating schools were predominantly located in lower SES areas (54%), were classified as large schools (55%), and were located in major cities (59%). See Table 1 for a summary of school characteristics.

**Table 1:** School characteristics (*N* = 160)

Number of student enrollments, mean^a^ (SD)	344 (320)
**Year range**	
Primary (5–2 years)	141 (88)
Combined (5–18 years)	19 (12)
**School sector, *n* (%) **	
Government	144 (90)
Independent	16 (10)
**Socioeconomic status^b^, *n* (%) **	
Lower socioeconomic status	87 (54)
Higher socioeconomic status	73 (46)
**School size^c^, *n* (%) **	
Small (<264 student enrolments)	72 (45)
Large (≥264 student enrolments)	88 (55)
**Geolocation^d^, *n* (%) **	
Major cities	94 (59)
Regional/remote^e^	66 (41)

a Student enrollment: the total number of students enrolled in the school.

b Socioeconomic status: school postcodes used to classify SES area based on 2016 Socio-Economic Index for Australia (SEIFA) [29]. The top 50% classified as high SES, and the lower 50% classified as low SES;

c School size: small <264, large ≥264;

d Geolocations as classified by the Australian Statistical Geography Standard.

e “rural/regional” includes very remote, remote, outer regional, inner regional.

### Reported barriers and facilitators to the adoption of SWAP IT

The four most frequently reported barriers to the adoption of SWAP IT were “expected workload for staff” (*n* = 52, 33%), “perception that parents and carers do not think it is the school’s place to provide this information” (*n* = 31, 19%), and “food insecurity is a greater priority for my community,” and “there are no barriers” reported by 8% of participants (*n* = 13). The three most frequently reported facilitators were “keep the program free” (*n* = 60, 38%), “show evidence that SWAP IT supports the development of healthy habits in children” (*n* = 18, 11%), and “make the registration process easy” (*n* = 12, 8%) (see Table 2).

**Table 2:** Primary barrier and facilitator to adopting SWAP IT

Primary barrier	*n* (%)
Expected workload for staff	52 (32.50)
Perception that parents and carers do not think it is the school’s place to provide this information	31 (19.38)
Food insecurity is a greater priority for my community	13 (8.13)
There are no barriers	13 (8.13)
Concerned about how the program is delivered	10 (6.25)
Already running health promotion programs, therefore, not needed	9 (5.63)
Lunchboxes are already healthy	8 (5.00)
Prefer not to say	8 (5.00)
Cultural barriers	7 (4.38)
Perception that the program is not suitable for all students	4 (2.40)
No support from the school	2 (1.25)
Lack of confidence, knowledge, or training in the area	1 (0.63)
Total “Other”	1 (0.63)
“Other” subcategories	
Cost	1 (0.63)

Primary facilitator	*n* (%)
Keep the program free	60 (37.50)
Show evidence that SWAP IT supports the development of healthy habits in children	18 (11.25)
Make the registration process easy	12 (7.50)
Prefer not to say	12 (7.50)
Support for the program from parents to carers	9 (5.63)
If it were mandatory	7 (4.38)
Endorsement by the education sector	7 (4.38)
Nothing would be helpful	6 (3.75)
Endorsement from other local schools	5 (3.13)
Support for the program from teachers	5 (3.13)
Alignment with the school plan and curriculum	5 (3.13)
Alignment with school health and wellbeing priorities	3 (1.88)
Endorsement/support by trusted health authorities	0 (0.00)
Total “Other”	11 (5.63)
“Other” subcategories	
Delivery support	6 (3.75)
Enabling parents to opt in or out	2 (1.25)
Ensure culturally and linguistically inclusive	1 (0.63)

### Associations between reported barriers and facilitators and school characteristics

Schools located in higher SES areas had 2.6 times the odds (95% CI: 1.3–5.2; *P = *.006) of identifying “expected workload for staff” to be the biggest barrier to adoption (Table 3). Schools located in lower SES areas had significantly greater odds (OR 2.4; 95% CI: 1.0–5.6; *P = *.04) of identifying “the perception that parents and carers do not think it is the school’s place to provide this information” as the most frequent barrier to adoption. No other school characteristics showed statistically significant associations with barriers to adoption.

**Table 3:** Associations between reported barriers and school characteristics

Barrier	*Expected workload for staff*		
	*n* (%)	Odds ratio (95% CI)	*P* value
**Socioeconomic status** ^a^			
Lower socioeconomic	20 (23.0)	–	.006
Higher socioeconomic	32 (43.8)	2.6 (1.3–5.2)	
**School size** ^b^			
Small	19 (26.4)	0.6 (0.3–1.2)	.14
Large	33 (37.5)	–	
**Geolocation** ^c^			
Major cities	33 (28.8)	1.3 (0.7–2.6)	.40
Regional/remote	19 (35.1)	–	

Barrier	*Perception that parents and carers do not think it is the school’s place to provide this information*		
	*n* (%)	Odds ratio (95% CI)	*P* value
**Socioeconomic status** ^a^			
Lower socioeconomic	22 (25.3)	2.4 (1.0–5.6)	.04
Higher socioeconomic	9 (12.3)	–	
**School size** ^b^			
Small	18 (25.0)	1.9 (0.9–4.3)	.11
Large	13 (14.8)	–	
**Geolocation** ^c^			
Major cities	14 (14.9)	0.5 (0.2–1.1)	.09
Regional/remote	17 (25.8)	–	

Barrier	*Food insecurity is a greater priority for my community*		
	*n* (%)	Odds ratio (95% CI)	*P* value
**Socioeconomic status^a^**			
Lower socioeconomic	9 (10.3)	1.9 (0.6–6.8)	.27
Higher socioeconomic	4 (5.5)	–	
**School size^b^**			
Small	9 (12.5)	3.0 (0.9–10.2)	.08
Large	4 (4.6)	–	
**Geolocation^c^**			
Major cities	7 (7.5)	0.8 (0.2–2.5)	.70
Regional/remote^d^	6 (9.1)	–	

a Socioeconomic status: school postcodes used to classify SES area based on 2016 Socio-Economic Index for Australia (SEIFA) [29]. The Top 50% classified as high SES, and the lower 50% classified as low SES.

b School size: small <264, large ≥264.

c Geolocations as classified by the Australian Statistical Geography Standard.

d “rural/regional” includes very remote, remote, outer regional, inner regional.

There were no statistically significant associations between identified facilitators and school characteristics (Table 4).

**Table 4:** Associations between reported facilitators and school characteristics

Facilitator	*Keep the program free*		
	*n* (%)	Odds ratio (95% CI)	*P* value
**Socioeconomic status** ^a^			
Lower socioeconomic	36 (41.4)	1.4 (0.8–2.8)	.27
Higher socioeconomic	24 (32.9)	–	
**School size** ^b^			
Small	32 (36.4)	1.1 (0.6–2.1)	.74
Large	28 (38.9)	–	
**Geolocation** ^c^			
Major cities	20 (30.3)	1.7 (0.9–3.3)	.11
Regional/remote^d^	40 (42.6)	–	

Facilitator	*Show evidence that SWAP IT supports the development of healthy habits in children*		
	*n* (%)	Odds ratio (95%, CI)	*P* value
**Socioeconomic status** ^a^			
Lower socioeconomic	11 (12.6)	1.4 (0.5–3.7)	.54
Higher socioeconomic	7 (9.6)	–	
**School size** ^b^			
Small	12 (13.6)	0.6 (0.2–1.6)	.30
Large	6 (8.3)	–	
**Geolocation** ^c^			
Major cities	8 (12.1)	0.9 (0.3–2.3)	.77
Regional/remote^d^	10 (10.6)	–	

Facilitator	*Make the registration process easy*		
	*n* (%)	Odds ratio (95%, CI)	*P* value
**Socioeconomic status^a^**			
Lower socioeconomic	5 (5.8)	0.5 (0.2–1.9)	.36
Higher socioeconomic	7 (9.6)	–	
**School size^b^**			
Small	7 (9.7)	1.8 (0.5–5.9)	.34
Large	5 (5.7)	**–**	
**Geolocation^c^**			
Major cities	6 (6.4)	0.7 (0.2–2.2)	.52
Regional/remote^d^	9 (9.1)	**–**	

a Socioeconomic status: school postcodes used to classify SES area based on 2016 Socio-Economic Index for Australia (SEIFA) [29]. The Top 50% classified as high SES, and the lower 50% classified as low SES.

b School size: small <264, large ≥264.

c Geolocations as classified by the Australian Statistical Geography Standard.

d “rural/regional” includes very remote, remote, outer regional, inner regional.

## Discussion

This study aimed to identify the key determinants (i.e. barriers and facilitators) influencing the adoption of an effective school-based nutrition program (SWAP IT) and the potential associations with school characteristics. The determinants identified will subsequently inform the selection and development of implementation and scale-up strategies used to encourage the widespread uptake of the program to maximize population-level health benefits. The findings indicated that more than one-third of key decision makers from schools reported “expected workload for staff” to be the primary barrier to adoption (33%), while “keeping the program free” (38%) emerged as the most frequently reported facilitator. These results suggest that the capacity of those responsible for program implementation, and the perceived burden and cost of program delivery, appear to be critical factors influencing adoption.

This study provides key insights to inform future approaches to scaling up school-based nutrition education programs, an area in which, to our knowledge, limited evidence currently exists. SWAP IT incorporates all three components of the World Health Organization HPS framework, and therefore, these findings may be transferable to similar multicomponent school-based nutrition interventions. Previous research has primarily focused on barriers and facilitators to the uptake of interventions targeting just one component, such as school processes and procedures, including the provision of school meals, and nutrition-related policies and guidelines [27]. Despite this, the most frequently identified determinants in this study (i.e. expected workload for staff and keeping the program free) broadly align with findings from previous systematic reviews that have examined school-based nutrition-related interventions, policies, and programs. For example, a 2023 systematic review reported that the most prevalent barriers and facilitators to implementing nutrition-related interventions, policies, and programs in school-based settings included cost, target group needs and resources, and structural resources [27]. Furthermore, an additional 2018 systematic review identifying barriers and facilitators to school-based health and well-being programs identified “environmental context and resources” and “social influences” to be the most frequently reported determinants to adoption [43]. Our findings reiterate these results and highlight the need for such factors to be considered when planning scale-up approaches, and the importance of aligning health promotion programs with contextual factors, such as school capacity and parental perceptions, to maximize population-level adoption.

This study also sought to examine potential associations between identified determinants and school characteristics. SES was the only school characteristic found to be significantly associated with barriers to adoption. Schools located in higher SES areas were more likely to identify “expected workload for staff” as the biggest barrier to adoption, whereas schools located in lower SES areas were more likely to identify “perception that parents and carers do not think it is the school’s place to provide this information” as the biggest barrier to adoption. Previous research has highlighted the need to ensure school-based nutrition programs are implemented in priority schools, such as those located in lower SES areas [42]. However, currently, limited evidence exists examining differences in determinants of adoption in such schools. Our findings suggest that tailoring the selection and content of scale-up strategies according to the socioeconomic characteristics of the school, may be necessary to ensure an equitable approach to scale-up within priority groups [44]. The *Adapting Strategies to Promote Implementation Reach and Equity* framework supports this approach to ensure alignment between implementation and scale-up strategies and key determinants to facilitate equitable adoption [44].

There were no statistically significant associations between the identified facilitators to program adoption and school characteristics, with all school types identifying “keeping the program free” as the primary facilitator. This suggests that employing strategies that emphasize the program being available for free may be effective in achieving maximal adoption at scale. Previous studies have typically identified determinants during the evaluation phase of health promotion programs. However, identifying barriers and facilitators prior to scale-up may increase the likelihood of broader reach and scale-up success. Identifying determinants throughout the scale-up planning phase can inform the selection and development of strategies to promote widespread program adoption, reach and coverage. The identified determinants can be mapped to implementation and scale-up strategies using the constructs of innovation adoption by Wisdom and colleagues [45]. During this process, scale-up strategies may be developed and selected to address each relevant construct, with definitions informed by the Expert Recommendations for Implementing Change (ERIC) taxonomy [46]. Additionally, behavior change techniques can also be incorporated into strategies based on the behavior change wheel [40]. Behavior change techniques ensure the use of specific techniques that have been shown to elicit change in the behavior of choice (i.e. adoption) [47]. Aligning implementation strategies with identified determinants can increase the success of scaling up to achieve population-level health outcomes [23]. Such tailoring can be achieved by modifying the behavior change techniques embedded within implementation strategies, ensuring they are responsive to the unique needs of different school contexts, facilitating equitable adoption [48].

### Limitations

Limitations of this study include the response rate of the schools that were invited to participate in the study and subsequent impacts on the generalizability of the findings. Nonetheless, the participation rate of this study is consistent with that of previous primary school-based surveys, which have ranged 40%–60% [7, 49–52]. An additional limitation to acknowledge is that the data collection tool required respondents to select a single primary barrier and a single primary facilitator to adoption, from just one key decision maker at each school. This allowed key decision makers to select the most significant determinant to adoption; however, it did not allow the ability to report multiple determinants. Future studies may consider ranking determinants to ensure that all key determinants are identified and look to capture multiple perspectives. A further study limitation is the small number of school characteristics examined. Additional school characteristics (such as staffing roles and indigenous enrolments) may also impact the equitable adoption of school-based nutrition programs. Importantly, contextual factors impacting the adoption of school-based nutrition programs are dynamic and have the potential to change over time. Therefore, continuous assessment of determinants throughout the implementation and scale-up of school-based nutrition programs should be considered.

## Conclusions

Schools are a key setting for implementing effective health promotion programs aimed at improving children’s dietary intake. To achieve population-level health impact, these programs must be implemented at scale. Identifying the determinants of program adoption, prior to scale-up, is essential to ensure a tailored and equitable approach is taken to increase the adoption of such programs. This study provides novel insights into the school determinants of the adoption of an evidence-based school nutrition program, as well as the associations with key school characteristics. These findings offer valuable insights into the contextual factors influencing program adoption and may help guide the selection of scale-up strategies to ensure equitable uptake of effective school-based nutrition programs to achieve healthier dietary habits among children.

## Data Availability

De-identified data from this study are not available in a public archive. The quantitative datasets used during this study are available from the corresponding author on reasonable request.

## Welfare of Animals

This study does not contain any studies with animals performed by any of the authors.

## Artificial intelligence

Artificial intelligence was not used in the development of this manuscript.
